# Transcriptional Response of Virus-Infected Cassava and Identification of Putative Sources of Resistance for Cassava Brown Streak Disease

**DOI:** 10.1371/journal.pone.0096642

**Published:** 2014-05-20

**Authors:** M. N. Maruthi, Sophie Bouvaine, Hale A. Tufan, Ibrahim U. Mohammed, Rory J. Hillocks

**Affiliations:** Natural Resources Institute, University of Greenwich, Chatham Maritime, Kent, United Kingdom; USDA-ARS-SRRC, United States of America

## Abstract

Cassava (*Manihot esculenta*) is a major food staple in sub-Saharan Africa, which is severely affected by cassava brown streak disease (CBSD). The aim of this study was to identify resistance for CBSD as well as to understand the mechanism of putative resistance for providing effective control for the disease. Three cassava varieties; Kaleso, Kiroba and Albert were inoculated with cassava brown streak viruses by grafting and also using the natural insect vector the whitefly, *Bemisia tabaci*. Kaleso expressed mild or no disease symptoms and supported low concentrations of viruses, which is a characteristic of resistant plants. In comparison, Kiroba expressed severe leaf but milder root symptoms, while Albert was susceptible with severe symptoms both on leaves and roots. Real-time PCR was used to estimate virus concentrations in cassava varieties. Virus quantities were higher in Kiroba and Albert compared to Kaleso. The Illumina RNA-sequencing was used to further understand the genetic basis of resistance. More than 700 genes were uniquely overexpressed in Kaleso in response to virus infection compared to Albert. Surprisingly, none of them were similar to known resistant gene orthologs. Some of the overexpressed genes, however, belonged to the hormone signalling pathways and secondary metabolites, both of which are linked to plant resistance. These genes should be further characterised before confirming their role in resistance to CBSD.

## Introduction

Cassava (*Manihot esculenta* Crantz, Family: *Euphorbiaceae*) is an important food staple for over 700 million people across the tropical and sub-tropical world. It can be grown all year round and provides valuable food in periods when other food staples are not available [1]. Cassava production in Africa is threatened by two viral diseases; cassava mosaic disease (CMD) and cassava brown streak disease (CBSD), each causing up to 100% damage in susceptible varieties and severely affecting the food security in the continent [2]. Several cassava mosaic begomoviruses (CMBs) cause CMD, which is controlled effectively through the identification and introduction of resistant varieties [2], [32]. CBSD has for many years been recognized as an important disease of cassava in coastal East Africa and the shores of Lake Malawi and Mozambique in the South [3], [4] but more recently the disease is expanding towards central Africa in parts of Uganda, Kenya, Tanzania, Burundi, Rwanda and Congo [2], [4]–[6]. CBSD is caused by two distinct species of single-stranded RNA (ssRNA) viruses, *Cassava brown streak virus* (CBSV) and *Ugandan cassava brown streak virus* (UCBSV), belonging to the genus *Ipomovirus* of the family *Potyviridae* [7], [8], [43]. Both viruses are transmitted by whiteflies, *Bemisia tabaci*, in a semi persistent manner [9], [10]. The virus can also be transmitted by grafting or mechanically under laboratory conditions [16], and the disease is also spread by propagating infected cuttings in the field.

The most effective approach to reducing losses due to CBSD is by deploying resistant cultivars. Early breeding efforts in coastal Tanzania made use of resistance introgressed into cultivated cassava from the close relative - Ceara Rubber (*Manihot glaziovii*) [11]. The most resistant hybrid developed from this programme was 46106/27, which is currently grown under the local name Kaleso in Kenya, and Namikonga in Tanzania [12]. More recent efforts in eastern Africa have identified a number of cassava varieties resistant/tolerant to CBSD, which can be infected with CBSVs but either show no, mild or delayed symptoms of root necrosis. They provide some relief against the disease while more resistant varieties with high yields are urgently required to minimise the impact of the disease on affected communities. The availability of procedures such as real-time quantitative PCR (RT-qPCR) for measuring virus titres accurately [13] and RNA-sequencing (RNA-Seq) technologies can make immediate impact in identifying resistance as well as unravelling the mechanism and genetic basis of resistance. RNA-Seq [14] in particular, produces millions of short cDNA reads that are mapped to a reference genome to obtain a genome-scale transcriptional map, which consists of the transcriptional structure and the expression level for each gene potentially contributing to virus resistance.

The aim of this study was first to characterize the level of resistance to CBSD of three cassava varieties Albert, Kiroba and Kaleso and second to compare the transcriptome of a resistant and a susceptible cassava to uncover transcripts putatively involved in disease resistance. The three varieties were challenged with CBSV and UCBSV by graft inoculation as well as by whitefly inoculation. Multiplication of viruses in plants was monitored by RT-qPCR and the severity of disease symptoms was recorded both on leaves and roots. Based on these parameters, variety Kaleso was considered to be resistant to CBSD because it had no or mild disease symptoms and low quantities of viruses. Variety Albert was considered to be susceptible as it developed severe symptoms and supported high levels of virus concentrations. Variety Kiroba was considered to be tolerant as it had intermediate type of symptoms and virus concentrations compared to Kaleso and Albert. In order to understand the mechanism of resistance, Kaleso and Albert were compared by RNA-Seq. Over 700 genes were found to be up-regulated in response to CBSV infection in the resistant variety Kaleso. The gene expression profiles of Kaleso and Albert are presented here as a first study towards identifying CBSD resistance genes.

## Materials and Methods

### Testing Cassava Varieties for Virus Resistance

Three cassava varieties Albert, Kiroba and Kaleso differing in response to CBSD were used in this study. Kaleso is a widely adopted variety in Kenya, and Kiroba and Albert are varieties grown in Tanzania [12], [15]. Plants were confirmed to be free of viruses by end-point reverse transcription polymerase chain reaction (RT-PCR) (see below) and they were generated by tissue culturing. Two virus isolates that differed in levels of severity were used to inoculate the cassava varieties; the severe isolate of CBSV was collected from Mozambique (CBSV-[MZ:Nam1-1∶07]), and the relatively milder isolate of UCBSV was from Uganda (UCBSV-[UG:Kab4-3∶07]) [16].

In order to characterise resistance to CBSD, the UCBSV or CBSV were graft-inoculated onto two-month-old healthy cassava plants of the three cassava varieties (five plants for each variety/virus combination). Plants were kept in relatively constant environment at 28±5°C and 50–60% RH for symptom development. Plants showing no symptoms or signs of virus infection when tested by RT-PCR after four weeks were inoculated again at four weekly intervals until all plants showed symptoms. The efficiency of graft transmission was calculated on each variety by the number of plants with leaf and root symptoms. CBSD symptoms on leaves and stems were recorded at four week intervals for nine months. Disease severity on roots was recorded about a year after graft-inoculation by visual inspection of roots. Roots were cut at 1 cm interval and scored on a 5-point scoring system in which 1 = no visible root symptoms, and 5 = very severe necrosis of affected roots (affects >30% of root surface) [15], [17]. A number of factors including the efficiency of graft-transmission, time taken for symptom expression and disease severity were recorded to confirm the levels of resistance to CBSD.

### Virus Detection by PCR

A single lobe of a fully expanded leaf (fourth or fifth from the top) and a small portion of roots (non-destructive sampling) were collected from the three cassava varieties for detecting CBSVs by RT-qPCR [18], [19]. Samples were taken at 24 h intervals after the grafting in the first week, subsequently at weekly interval for four weeks, followed by monthly interval for nine months. Thirty-six samples were collected at each time point for each variety-virus combination (3 varieties×2 virus isolates×3 plants×2 samples). A total of 540 samples were collected by the end of nine months and analysed for the presence of the virus by RT-PCR [18], [19] and quantified subsequently by RT-qPCR on a subset of samples. The subset of samples was taken from plants confirmed to be infected with viruses by RT-PCR. Total nucleic acids were extracted from samples using a protocol described before [19]. RNAs selected for virus quantification were DNAse treated according to the manufacturer conditions (DNase RQ 1 treatment kit, Promega, USA) and the concentration was estimated using a Nanodrop 2000 (Thermo Scientific Ltd., UK). Approximately 1 µg of RNA was used for cDNA synthesis using ImProm-II Reverse Transcriptase kit (Promega, UK) and random primers (New England Biolabs, UK). Virus detection was performed in 96 well-plates; each reaction was carried out in 25 µl reaction containing 1x QuantiTect SYBR Green (Qiagen), 7.5 nM primers (Table S1) and 2.5 µl cDNA. The Master Cycler Ep Realplex PCR (Eppendorf, UK) was used at an initial 15 min at 95°C then 40 cycles of 94°C for 15 sec, 52°C for 30 sec and extension at 72°C for 30 sec. Melting curve analysis was carried out subsequently on three technical replicate samples to confirm the specificity of the reaction. To minimize errors due to pipetting, dispensing of reagents for RT-qPCR and cDNA synthesis was carried out in a robot EpMotion 5070 (Eppendorf, UK). The virus cDNA detected was normalized to the expression of the cassava gene ribulose biphosphate carboxylase oxygenase gene (RubiscoL) (Table S1) using the 2^−ΔΔCt^ method [20].

### Virus Inoculation by Whiteflies, and Whitefly Preference for Cassava Varieties

In order to estimate the level of virus transmission to three cassava varieties by whiteflies, approximately 1000 adult *B. tabaci* were collected from a whitefly colony originated from Uganda [21], [22]. These were introduced into a cage (60×60×90 cm) containing three two-month-old CBSV infected cassava plants of var. Ebwanateraka for 24 h. About 30 *B. tabaci* were then transferred onto each two-month-old healthy cassava plants for 24 h for virus inoculation [21]. Thirty plants were inoculated per replication for each variety and three replications were included in the experiment to give a total of 90 inoculated plants. The *B. tabaci* were removed manually after inoculation, and plants were maintained for three months at 28±5°C and 50–60% RH. The susceptibility of the varieties was determined by RT-PCR based on the proportion of infected plants three months after virus inoculation.

In order to estimate the resistance of cassava to whiteflies, five male and female insects were transferred into a clip cage and allowed to feed on three cassava varieties [22]. Fifty such cages were set up separately for each variety in three replicates. Whiteflies were allowed to lay eggs for 48 h and then removed. Plants were kept at 28±5°C, 50–60% RH and L12:D12. The number of eggs laid, nymphs developed and adults emerged were recorded on each variety at weekly interval for up to four weeks. Data obtained were analysed by ANOVA to determine the effect of varieties on the fecundity and survival of *B. tabaci*.

### Transcriptome Analysis using RNA-Seq Illumina Sequencing

RNA-Seq was carried out to understand the mechanism of CBSD resistance in cassava. Leaf samples were collected from three CBSV-inoculated and control (un-inoculated) plants of Albert and Kaleso (three biological replicates) one year after graft inoculation with CBSV. At this time point, all plants still showed symptoms and tested positive for the viruses by RT-PCR. Total nucleic acid was isolated from 100 mg of cassava leaf tissue as described before [19] except that these samples were ground using liquid nitrogen. To isolate total RNA, 1 µg of the sample was then DNAse treated using RQ1 DNAse (Promega, USA). Samples were concentrated and cleaned up using RNEasy MinElute Clean up kit (Qiagen, Germany). Resulting RNA was quantified on Nanodrop and quality was checked on Agilent 2100 Bioanalyzer (Agilent, USA). Equimolar quantities of each of three biological replicates for each variety were pooled at this stage. cDNA libraries and RNA-Seq were performed by GATC Biotech (Konstanz, Germany) for generating 50 bp single end reads using Illumina Hiseq 2000 platform. The raw sequences were submitted to NCBI’s Gene Expression Omnibus database (GEO), which were assigned a series entry accession number GSE56467. Sequences are available for public use from the website http://www.ncbi.nlm.nih.gov/geo/query/acc.cgi?acc=GSE56467.

Sequence reads were mapped against the cassava genome retrieved from Phytozome (http://www.phytozome.net/cassava.php) using BWA aligner [23]. Only uniquely mapped reads were retained. The alignments were processed to compute the read counts for each transcript. The expression for each gene was generated as read per kilobase per million reads (RPKM). Only transcripts showing RPKM values>1 in at least one sample were kept for downstream analysis. Differential expression between two samples was assessed using the statistical R package DEGseq [24] using a MA-plot-based method and a random sampling model. Genes were filtered at a level of 2-fold or greater difference between two samples. The homologs of differentially expressed genes were queried in The Arabidopsis Information Resource (TAIR) functional categorization tool (http://www.arabidopsis.org/tools/bulk/go/index.jsp). The Kyoto Encyclopaedia of Genes and Genomes (KEGG) database is extensively used to assign metabolic pathways to genes [25]. Using TAIR homologues, the enrichment of KEGG pathways in overexpressed genes was calculated using the Database for Annotation, Visualization and Integrated Discovery (DAVID) [26], [27]. For validating RNA-Seq data, primers were designed for selected overexpressed genes of interest using the NCBI-primer blast tool (Table S1). Gene expression was measured by RT-qPCR, in the conditions indicated above in three biological replicates of infected and healthy cassava plants. The amplification of selected genes was normalized to the expression of the cassava gene ribosomal protein (L2) (Table S1) using the 2^−ΔΔCt^ method [20]. The L2 primers were designed from a BLAST search of the cassava genome on the website Phytozome. Efficiency of primers was tested and primers displaying an efficiency of at least 0.85 were retained.

## Results

### The Response of Varieties for Virus Infection by Graft Inoculation

Four weeks after the first graft inoculation with CBSV or UCBSV, none of the five Kaleso plants showed CBSD symptoms. The symptoms of Kiroba and Albert varied between the two viruses. All five plants of Albert were infected with CBSV within four weeks of virus inoculation. For Kiroba, only two were infected in the same period (Table 1). Four independent grafts were required to infect all plants of Kaleso with CBSV. In comparison, all five plants of Kiroba were infected by the second graft. In general, CBSV infected cassava varieties quicker and more efficiently than UCBSV. Plants infected with CBSV also exhibited severe symptoms compared to the milder symptoms of UCBSV. With regards to root necrosis, based on a scale of 1 (no symptoms) to 5 (very severe symptoms) [15], [28] all plants of Albert showed symptoms with a severity score of 3.0 for UCBSV and 4.0 for CBSV, while an average symptom score of 1.5 was recorded for Kiroba plants for both viruses and had a maximum score of 2.0 for each virus. None of the Kaleso roots showed signs of damage for both viruses except for faint discolouration at the pith by CBSV.

**Table 1:** Number of plants infected with CBSV or UCBSV for the three cassava varieties during the time course of virus transmission by grafting.

			No. of plants infected/no. of grafts made		Percentage of plants infected	
Cassava varieties	No. of graftsdone	Time since1^st^ graft (weeks)	UCBSV	CBSV	UCBSV	CBSV
Kaleso	1st	0	0/5	0/5	0	0
	2nd	4	0/5	3/5	0	60
	3rd	8	2/5	2/2	40	100
	4th	12	3/3	-	100	-
Kiroba	1st	0	0/5	2/5	0	40
	2nd	4	2/5	3/3	40	100
	3rd	8	3/3	-	100	-
Albert	1st	0	4/5	5/5	80	100
	2nd	8	1/1	-	100	-

- grafting was not done since all plants expressed symptoms from the previous grafting at this time point.

### Virus Detection and Spread in Cassava Varieties

CBSV and UCBSV were not detected in any plants of the three varieties 24 h and 48 h after inoculation. CBSV was first detected four days after inoculation in the roots of one of the three plants of Albert, while none of the Kaleso and Kiroba plants tested positive for either virus at this time point (Table 2). Both viruses were detected from leaves and roots of Albert at one week after virus inoculation, and in comparison it took 2–8 weeks for detecting viruses in all plants of Kiroba and Kaleso. At 12 weeks, all plants of all varieties showed the presence of viruses in both leaves and roots. Some plants of Kaleso and Kiroba that were shown to be infected with viruses early reacted negative for viruses by RT-PCR in the middle of the experiment, but they again reacted positively towards the end. This is indicating recovery of cassava plants from virus infections, or localised infections of viruses in any given plant tissue of these two varieties. In comparison, both viruses were detected consistently in Albert for up to 36 weeks.

**Table 2:** Number of cassava plants infected with CBSV or UCBSV in roots and leaves over a period of 12 weeks after virus inoculation by grafting as confirmed by RT-PCR.

	Number of plants with UCBSV (out of 3)						Number of plants with CBSV (out of 3)					
	Kaleso		Kiroba		Albert		Kaleso		Kiroba		Albert	
Hours/Weeks	leaf	root	leaf	root	leaf	root	leaf	root	leaf	root	leaf	root
24 h	0	0	0	0	0	0	0	0	0	0	0	0
48 h	0	0	0	0	0	0	0	0	0	0	0	0
96 h	0	0	0	0	0	0	0	0	0	0	0	1
1	0	0	0	0	1	1	0	0	0	0	1	2
2	0	1	0	2	0	2	0	0	1	2	1	2
4	0	2	0	0	1	3	0	0	1	2	3	3
8	2	2	3	2	3	3	2	1	1	2	3	3
12	3	3	3	3	3	3	3	3	3	3	3	3

**Figure 1:**
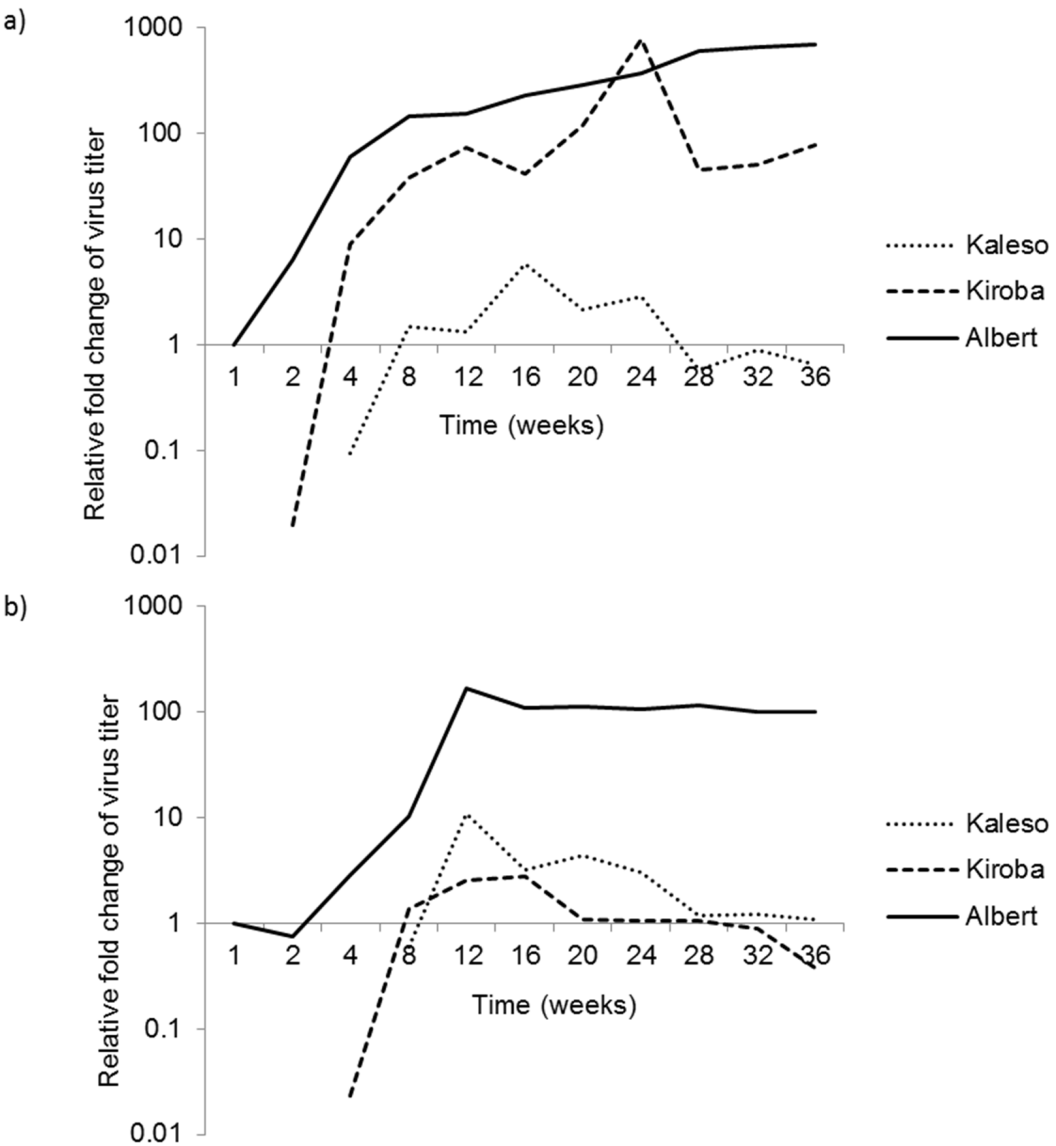
Relative changes of virus titre in cassava plants for a) CBSV and b) UCBSV. Virus quantities were normalised to the concentration of virus detected in Albert at the first week time point.

Quantification of CBSV and UCBSV in cassava varieties indicated significant differences in virus concentrations among the cassava varieties (Friedman test, CBSV: χ^2^ = 19.6, p<0.001, UCBSV: χ^2^ = 18.476, p<0.001). Albert showed highest levels of virus concentration compared to Kiroba and Kaleso, the latter two showed medium to very low levels, respectively (Figure 1). Concentration of both viruses did not vary considerably throughout the sampling period in Kaleso as they remained very low, while they were high in concentration in Albert. The concentration of UCBSV increased over 160-fold from first week up to 16 weeks in Albert and the virus multiplication seem to have stabilised for the remainder of the experiment up to 36 weeks. Kiroba displayed peak CBSV and UCBSV concentrations at 16 and 24 weeks, respectively. Concentration UCBSV was marginally higher than CBSV in Kiroba, although they both decreased subsequently and remained low for the rest of the experiment. In comparison, the concentration of CBSV increased up to 700 times in Albert and the virus was still actively multiplying at 36 weeks.

### Whitefly Survival on Cassava Varieties and Virus Transmission

**Figure 2:**
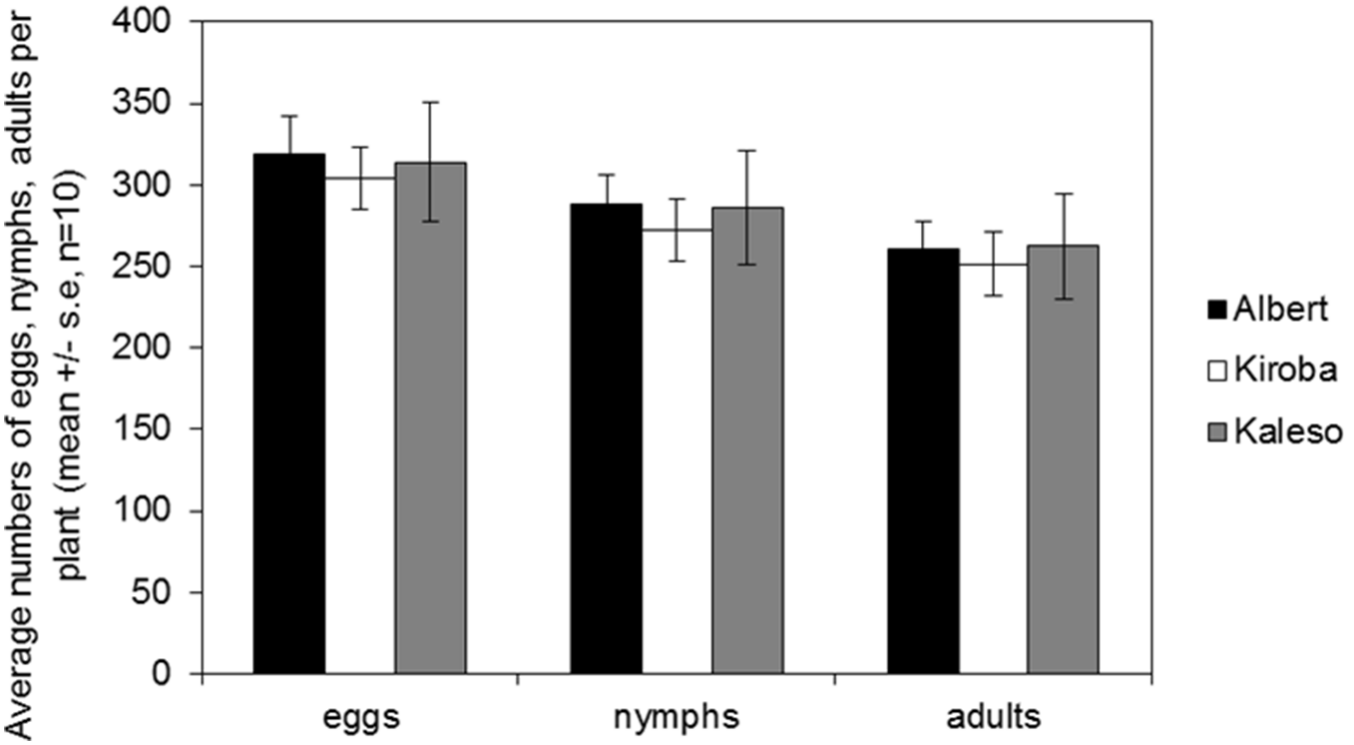
Average number of eggs, nymphs and whitefly adults recorded on the three cassava varieties after five female and male insects were allowed to feed and develop for one life cycle of 30 days.

In order to understand resistance levels of cassava varieties to CBSD in natural conditions where the virus is transmitted by the vector *B. tabaci*, cassava plants were assessed for their resistance to whiteflies. All three cassava varieties equally supported whitefly egg laying and reproduction (Figure 2). Minor differences observed in the numbers of eggs laid (F_2,27_ = 0.078, p = 0.925), nymphs developed (F_2,27_ = 0.111, p = 0.896) and adults emerged (F_2,27_ = 0.059, p = 0.942) were not statistically significant between the three cassava varieties. The percentage of eggs that survived to nymphs across the varieties ranged from 90–91%, nymphs to adults 91–92% and eggs to adults survival 82–84%. These differences in development of *B. tabaci* stages among cassava varieties from eggs to nymphs, nymphs to adults and eggs to adults were also not significant (P>0.05).

In the whitefly transmission experiment, about 57% and 47% of Albert and Kiroba plants developed CBSD symptoms, respectively, while only one of 30 Kaleso plants (3%) was infected. The differences in CBSV infection among cassava varieties was highly significant (ANOVA, F_2,6_ = 24.1, P<0.001). The number of weeks required from inoculation to symptom appearance varied; in Albert the first plant showed symptoms three weeks after inoculation by *B. tabaci* while in Kiroba and Kaleso, first symptoms appeared five and eight weeks after inoculation, respectively. Symptoms on whitefly-transmitted plants were milder to those seen on wild-type plants possibly because of growing the plants in plastic bags in artificial insectary conditions.

### RNA-Seq Analysis of Gene Expression

The two cassava varieties Albert and Kaleso were chosen for transcriptome analysis by Illumina RNA-Seq as they showed the most contrasting phenotype for CBSV susceptibility. RNA-Seq was conducted one year after virus inoculation to understand the steady state of response to virus infection. Between 49 and 60 million raw sequencing reads were generated from four samples; Kaleso CBSV-infected and control (healthy), and Albert CBSV-infected and control. Read counts, reads per million mapped sequence reads and RPKM values were generated for each gene in the cassava reference genome. Approximately 60% of the reads were uniquely mapped to the genome (21 to 36 millions), which represented 22,368 genes expressed in at least one sample with an RPKM value >1 (Table 3). The remaining genes, showing RPKM values below one across all treatments, were excluded from further analysis. The quality of the sequences obtained was considered good as the number of highly expressed genes in Kaleso control, Albert control and Albert CBSV treatments was around 100, while the number of genes highly expressed specifically in Kaleso CBSV treatment was markedly less at 67.

**Table 3:** Number of reads generated from the RNA-Seq analysis and the corresponding gene expression range obtained for resistant Kaleso and susceptible Albert cassava varieties.

	Albert healthy	Albert CBSV	Kaleso healthy	Kaleso CBSV
All reads	54,045,667	60,070,579	38,949,010	49,681,907
Mapped to whole genome	31,632,660	35,964,664	20,946,755	29,534,087
**Number of genes with:**				
RPKM>1000	105	102	102	67
RPKM>100	2,150	2,246	2,225	2,337
RPKM>10	12,656	12,628	13,268	13,801
RPKM>1	20,185	20,071	20,686	21,224

**Figure 3:**
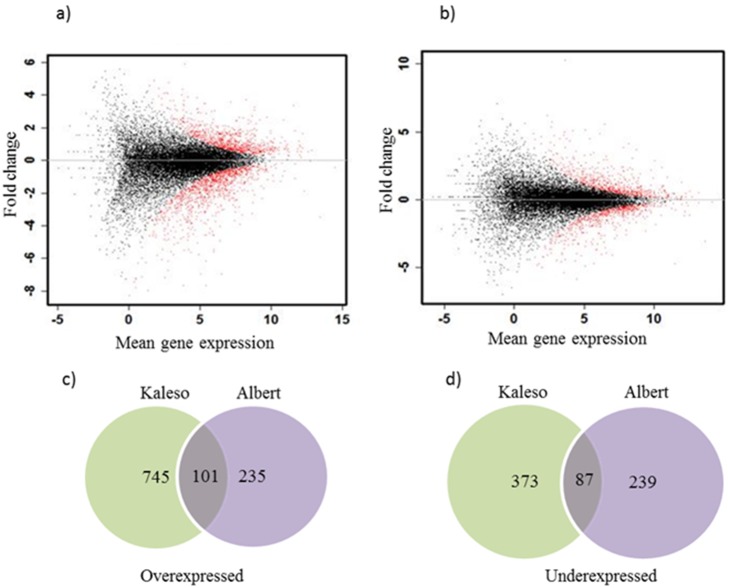
M/A plots of the expression of all genes of healthy vs infected cassava a) Kaleso and b) Albert. The red dots indicate the significantly differentially expressed genes. c) Venn diagram of the significantly over- and under-expressed genes.

Among the top 10 highly expressed genes, nine were common in all samples, those genes included three ribulose biphosphate carboxylases, two calcium binding family proteins, one photosystem II subunit and other un-annotated transcripts (Table S2). Using the random sampling model in the DEGseq program, pairwise comparisons of gene expression were carried out between the infected and non-infected samples of Kaleso and Albert (Table S3). Mapped read count for each gene with a p-value<0.001 were obtained, and the MA-plot revealed little variation between the infected and non-infected samples (Figure 3a and b).

In response to CBSV infection, a total of 1081 genes were significantly overexpressed at least two-fold in the two cassava varieties. Among those genes, 745 were unique to Kaleso, 235 were unique to Albert while 101 were equally overexpressed in both varieties (Figure 3c and d). Among the 745 genes, 130 were at least 10 times overexpressed in Kaleso in response to CBSV infection but only 25 in Albert. A smaller number of genes were under expressed (373 uniquely in Kaleso and 239 in Albert).

Among all the genes overexpressed in Kaleso (uniquely + commonly with Albert = 846), 784 were found to have orthologs in *Arabidopsis thaliana*. The orthologs were then used to find gene ontology and pathways enriched in the plant using the TAIR database. Analysis of gene ontology revealed a large number of genes involved in stress signalling that were overexpressed in both varieties (Table S1). No differences in genes assigned to GO categories however could be identified between the two varieties. The up-regulated genes in Kaleso were then assigned to biochemical pathways in the KEEG database using the DAVID bioinformatics tool. Six pathways were found to be enriched among the overexpressed genes in Kaleso (Table 4, Table S4), representing 99 genes. Interestingly, 20 of these were assigned to the plant hormone signalling pathways, indicating a potential elevation of plant hormones in Kaleso plants. In addition to primary metabolite pathways such as fructose and mannose metabolism and amino sugar and nucleotide sugar metabolism, three pathways were found to be involved in the synthesis of secondary metabolites: biosynthesis of phenylpropanoids (20 genes), terpenoids and steroids (16 genes), and flavonoids (6 genes).

**Table 4:** KEGG pathways up-regulated in the resistant cassava variety Kaleso as determined by DAVID bioinformatics tool.

KEGG pathway number	Term	Number ofgenes	% of totalgenes present in the pathway	P-Value
ath01061	Biosynthesis of phenylpropanoids	20	3	7.8E-3
**ath01070**	Biosynthesis of plant hormones	20	3	5.7E-2
**ath01062**	Biosynthesis of terpenoids and steroids	16	2.4	1.6E-2
ath00520	Amino sugar and nucleotide sugar metabolism	10	1.5	3.5E-3
ath00051	Fructose and mannose metabolism	8	1.2	2.7E-3
**ath00680**	Methane metabolism	8	1.2	5.7E-2
ath03050	Proteasome	7	1	3.7E-2
ath00941	Flavonoid biosynthesis	6	0.9	9.7E-4
ath00906	Carotenoid biosynthesis	4	0.6	6.6E-2

Manual analysis of 13 genes that were overexpressed at least 100 times in Kaleso identified a NAC-domain protein annotated transcript (cassava4.1_026167m). This transcript was overexpressed 140 times in CBSV infected Kaleso but poorly in healthy control and virus-infected Albert samples with RPKM values <1. Further analysis of the values highlighted that five other NAC proteins, homologs to three different TAIR10 NAC-proteins, were overexpressed in Kaleso 24 to 139 times in the presence of CBSV (Table 3). These genes were also overexpressed in Albert although only 2 to 21 times. Analysis by RT-qPCR further confirmed the overexpression of cassava4.1_026167m in Kaleso while it was poorly expressed in Albert (Table 5). RT-qPCR detected larger differences in expression for two NAC homologs than RNA-Seq (cassava4.1_015961m, cassava4.1_028212m) and for two others (cassava4.1_011029m, cassava4.1_023870m) the differences were markedly low, while one of the NAC-domain containing protein homolog could not be detected by RT-qPCR in either variety (cassava4.1_026590m) (Table 5).

**Table 5:** Fold enrichment of NAC-protein genes in infected cassavas as measured by RNA-Seq and RT-qPCR.

		Albert		Kaleso	
cassava gene-id	TAIR-10 ortholog	RNA-Seq	RT-qPCR	RNA-Seq	RT-qPCR
**cassava4.1_026167m**	AT5G22380.1	**undetected**	0.48	**140.4316**	71.5
**cassava4.1_026590m**	AT5G61430.1	**3.57374071**	undetected	**139.0273**	undetected
**cassava4.1_015961m**	AT4G35580.3	**21.3148107**	15.3	**72.8489**	204.4
**cassava4.1_028212m**	AT4G35580.3	**18.6983219**	177	**55.47049**	426
**cassava4.1_011029m**	AT5G46590.1	**12.861311**	0.21	**36.09093**	5.52
**cassava4.1_023870m**	AT4G35580.2	**2.20586065**	undetected	**24.51312**	2.67

### Identifying known Resistance Gene Analogues in Cassava

Several dominant resistance gene analogues (RGAs) conferring resistance to plant viruses have been identified in other crop and model species; *Nicotiana tabaccum, Solanum tuberosum, S. thaliana* and *S. lycopersicum* (for a review see [29]). The expression profiles of Kaleso and Albert were compared to these RGAs using common elements found among resistant genes such as an N-terminal domain with either a Toll interleukin-1 receptor (TIR) or a coiled domain (CC), a nucleotide binding site (NBS) or a leucine-reach repeat domain (LRR) [30]. Among the 235 genes with either NBS or LRR domains expressed in our experiments, none of them showed significant differences in expression as a result of virus infection (Table S5). In addition to NBS-LRR genes, cassava genome was screened for homologs of several other known RGAs. These include Tm-1 genes conferring resistance to *Tomato mosaic virus* and RTM-1, RTM-2 and RTM-3 conferring resistance to *Tobacco etch virus* in *A. thaliana*. In cassava, RGAs of RTM-2 and Tm-1 were found to be expressed but not differentially between the resistant and susceptible cassava varieties (Table S5).

In addition to dominant genes, recessive genes also contribute to plant resistance (for a review see [31]), which all encode translation initiation factors eIF4E, eIF4G or their isoforms. Analysis of cassava transcript profiles identified the expression of four eIF4E and one eIF4G. Amongst these, eIF4E transcript cassava4.1_016601m was increased two-fold in infected Kaleso compared to healthy and Albert controls (Table S2).

## Discussion

This study was initiated with the aim of characterizing the level of resistance to CBSD in three cassava varieties and understanding the transcriptomic response of resistant and susceptible cassavas when inoculated with CBSVs. The three varieties were inoculated with CBSV and UCBSV by whiteflies as well as by grafting. Virus inoculation by grafting has been perceived to be a stringent test for identifying resistance to CBSD since it introduces a high dose of virus particles directly into the phloem tissues of test plants and thus bypasses many inherent mechanisms of plant resistance such as hypersensitive reaction seen in leaves or resistance to the whitefly vector of the virus. Graft inoculation of three cassava varieties Kaleso, Kiroba and Albert in this study, however, showed to be highly effective for screening for CBSV and UCBSV resistance as the reaction of each variety varied and it was as expected. Kaleso was resistant to both CBSV and UCBSV as it took up to four repeat graft inoculations to infect all plants. In contrast, most plants of the susceptible variety Albert were infected in the first inoculation itself. The symptom severity and the time interval between virus inoculation and symptom appearance also varied significantly between the resistant Kaleso and susceptible Albert. Similar results were obtained in whitefly transmission experiment, as only 3% Kaleso plants were infected with CBSV compared to 47 and 57% of Kiroba and Albert, respectively. Put together, these results indicated that inoculation of CBSVs by grafting is suitable and reliable for screening for CBSD resistance in cassava. The current method of screening depends on the inoculation of virus by whiteflies in the field, which is not always reliable because the whitefly numbers vary greatly from season to season and location to location. Inoculation by grafting on the other hand is accurate and can yield quick results as there will be no ‘escapes’ due to low or no whiteflies in the field. Importantly, the time of virus infection will be known which is critical for subsequent RT-qPCR studies to determine virus load. These methods can also be used to eliminate the discrepancies and the confusion that exist in describing the levels of resistance for CBSD. This arise mainly because of the subjective nature of scoring CBSD symptoms visually and is further compounded by a range of symptoms seen on different cassava varieties [8], [16] as well as the lack of strict correlation between foliar and root symptoms on some varieties [4]. Virus inoculations by grafting and subsequent virus quantification by RT-qPCR are therefore most reliable for identifying CBSD resistance in cassava.

Many terminologies have been used inconsistently in the literature to describe response of cassava to CBSD, and in general of plants to virus infections [40]. Among them, immunity, resistant, tolerant and susceptible are most common. Immunity indicates non-infection of a plant by a given virus (non-host), which is not recorded for CBSVs in this or other studies [32], [33]. In the case of resistant plants, infection by viruses can occur but multiplication and movement is restricted, and the disease symptoms are generally localised or absent [40], [41]. These are the characteristics seen on Kaleso for CBSD in our studies and thus can be considered resistant. The term tolerance is used to describe a host that can be infected by a virus and in which it can replicate and invade without causing severe symptoms or greatly diminishing plant growth or yield [40]. Severe symptoms were seen on the leaves of Kiroba [16], but the titres of both CBSV and UCBSV were comparatively low. This variety was also released for cultivation in disease endemic areas of Tanzania because root symptoms were rare and thus the yield was not affected [34]. Kiroba can therefore fit with the description of a tolerant variety for CBSD. Albert is susceptible to CBSD as it expressed severe symptoms both on leaves and roots, and the virus multiplication and spread were unabated. The concentrations of viruses and the severity of symptoms seen on these three varieties can be used as a guide to describe the resistance levels of other cassava germplasm for CBSD.

The transcriptome analysis of the most resistant and susceptible varieties Kaleso and Albert was carried out to identify the mechanism of resistance and putative CBSD resistance genes. Transcriptome analysis was carried out 12 months after graft inoculation specifically to study genes involved in steady state defence responses, rather than early response genes [35]. Although independent analysis of biological replicates is important to draw sound conclusions on biological differences between responses of different cassava varieties to CBSV, the high cost of RNA-Seq analysis limited the number of samples analysed in this study. Pooling biological replicates for RNA-Seq has been previously utilized to draw conclusions on differential gene expression in plant systems [36], and would be sufficient for a snap-shot view of gene expression profiles long after virus inoculation, which were targeted in this study.

Analysis of differentially expressed genes demonstrated that the two cassava varieties had unique response to infection, with the resistant cassava showing the highest number of genes overexpressed. One family of proteins was confirmed to be overexpressed both by RNA-Seq and RT-qPCR in Kaleso in response to infection. NAC proteins constitute one of the largest families of plant transcription factors [37]. The expression of these genes has been demonstrated to be induced by biotic and abiotic stresses. It is of particular interest that these proteins have been identified as being able to bind specifically to viruses in wheat and *Arabidopsis* [38], [39]. Although the GO analysis did not reveal any category of gene up-regulated specifically in Kaleso but genes of importance in the hormone signalling pathway were overexpressed. In addition to phyotohormones, Kaleso also had elevated levels of transcripts involved in the synthesis of secondary metabolites such as terpenoids, steroids, flavonoids and phenylpropanoids. Secondary metabolites have important role in plant defence pathway as demonstrated with brassinosteroids [42]. The lack of up-regulation of known RGAs in cassava, which are the dominant genes identified conferring resistance to plant viruses, is probably not entirely surprising because CBSD resistance was considered to be multigeneic [11], possibly controlled by many recessive genes. The over expression of eIF4E transcript further support this hypothesis although their role in CBSD resistance remains to be confirmed. Further validation studies are also required to confirm the role of NAC proteins, hormone signalling pathways and secondary metabolites in the resistance of Kaleso to CBSD. Future efforts to identify CBSD resistant genes can be speeded up from using a combination of technologies such as RNA-Seq on more resistant varieties together with field breeding (crossing) between resistant and susceptible parents and mapping segregating populations by quantitative trait loci.

## Supporting Information

Table S1 **Sequence of primers used in this study for real time PCR.**
(DOCX)

Table S2 **All expressed genes detected by RNA-Seq in healthy and CBSV infected susceptible Albert and resistant Kaleso cassava varieties. The ten most expressed genes are highlighted in red.**
(XLSX)

Table S3 **Genes differentially expressed between healthy and infected susceptible Albert and resistant Kaleso cassava varieties.**
(XLSX)

Table S4 **Enriched KEGG pathways in the overexpressed genes in resistant cassava variety Kaleso.**
(XLSX)

Table S5 **Expression values of genes with NBS or LRR motifs.**
(XLSX)

## References

[1] Hillocks RJ, Thresh JM, Belloti A (2002) Cassava: Biology, Production and Utilization: CABI Publications.

[2] Legg JP, Jeremiah SC, Obiero HM, Maruthi MN, Ndyetabula I, et al (2011) Comparing the regional epidemiology of the cassava mosaic and cassava brown streak virus pandemics in Africa. Virus Research 159: 161–170. 21549776 10.1016/j.virusres.2011.04.018

[3] Nichols RF (1950) The brown streak disease of cassava; distribution climatic effects and diagnostic symptoms. East African Agriculture 15: 154–160.

[4] Hillocks RJ, Jennings DL (2003) Cassava brown streak disease: A review of present knowledge and research needs. International Journal of Pest Management 49: 225–234.

[5] Alicai T, Omongo CA, Maruthi MN, Hillocks RJ, Baguma Y, et al (2007) Re-emergence of Cassava Brown Streak Disease in Uganda. Plant Disease 91: 24–29. 10.1094/PD-91-0024 30781061

[6] Mulimbi W, Phemba X, Assumani B, Kasereka P, Muyisa S, et al (2012) First report of Ugandan cassava brown streak virus on cassava in Democratic Republic of Congo. New Disease Reports 26: 11.

[7] Monger WA, Alicai T, Ndunguru J, Kinyua ZM, Potts M, et al (2010) The complete genome sequence of the Tanzanian strain of Cassava brown streak virus and comparison with the Ugandan strain sequence. Archives of Virology 155: 429–433. 20094895 10.1007/s00705-009-0581-8

[8] Winter S, Koerbler M, Stein B, Pietruszka A, Paape M, et al (2010) Analysis of cassava brown streak viruses reveals the presence of distinct virus species causing cassava brown streak disease in East Africa. Journal of General Virology 91: 1365–1372. 20071490 10.1099/vir.0.014688-0

[9] Maruthi MN, Hillocks RJ, Mtunda K, Raya MD, Muhanna M, et al (2005) Transmission of *Cassava brown streak virus* by *Bemisia tabaci* (Gennadius). Journal of Phytopathology 153: 307–312.

[10] Mware B, Narla RD, Amata R, Olubayo F, Songa J, et al (2009) Efficiency of cassava brown streak virus transmission by two whitefly species in coastal Kenya. General and Molecular Virology 1: 40–45.

[11] Jennings DL (1960) Observations on virus diseases of cassava in resistant and susceptible varieties. II. Brown streak disease. Empirical Journal of Experimental Agriculture 28: 261–269.

[12] Ferguson ME, Hearne SJ, Close TJ, Wanamaker S, Moskal WA, et al (2012) Identification, validation and high-throughput genotyping of transcribed gene SNPs in cassava. Theoretical and Applied Genetics 124: 685–695. 22069119 10.1007/s00122-011-1739-9

[13] Adams IP, Abidrabo P, Miano DW, Alicai T, Kinyua ZM, et al (2013) High throughput real-time RT-PCR assays for specific detection of cassava brown streak disease causal viruses, and their application to testing of planting material. Plant Pathology 62: 233–242.

[14] Mortazavi A, Williams BA, McCue K, Schaeffer L, Wold B (2008) Mapping and quantifying mammalian transcriptomes by RNA-Seq. Nature Methods 5: 621–628. 18516045 10.1038/nmeth.1226 PMC13303166

[15] Hillocks RJ, Raya MD, Mtunda K, Kiozia H (2001) Effects of Brown Streak Virus Disease on Yield and Quality of Cassava in Tanzania. Journal of Phytopathology 149: 389–394.

[16] Mohammed IU, Abarshi MM, Muli B, Hillocks RJ, Maruthi MN (2012) The Symptom and Genetic Diversity of Cassava Brown Streak Viruses Infecting Cassava in East Africa. Advances in Virology 2012. 10.1155/2012/795697 PMC3290829 22454639

[17] Hillocks RJ, Raya M, Thresh JM (1996) The association between root necrosis and above-ground symptoms of brown streak virus infection of cassava in southern Tanzania. International Journal of Pest Management 42: 285–289.

[18] Abarshi MM, Mohammed IU, Jeremiah SC, Legg JP, Kumar PL, et al (2012) Multiplex RT-PCR assays for the simultaneous detection of both RNA and DNA viruses infecting cassava and the common occurrence of mixed infections by two cassava brown streak viruses in East Africa. Journal of Virological Methods 179: 176–184. 22080852 10.1016/j.jviromet.2011.10.020

[19] Abarshi MM, Mohammed IU, Wasswa P, Hillocks RJ, Holt J, et al (2010) Optimization of diagnostic RT-PCR protocols and sampling procedures for the reliable and cost-effective detection of Cassava brown streak virus. Journal of Virological Methods 163: 353–359. 19879299 10.1016/j.jviromet.2009.10.023

[20] Livak KJ, Schmittgen TD (2001) Analysis of relative gene expression data using real-time quantitative PCR and the 2(-Delta Delta C(T)) method. Methods 25: 402–408. 11846609 10.1006/meth.2001.1262

[21] Maruthi MN, Colvin J, Seal S, Gibson G, Cooper J (2002) Co-adaptation between cassava mosaic geminiviruses and their local vector populations. Virus Research 86: 71–85. 12076831 10.1016/s0168-1702(02)00051-5

[22] Maruthi MN, Colvin J, Seal SE (2001) Mating compatibility, life-history traits, and RAPD-PCR variation in *Bemisia tabaci* associated with the cassava mosaic disease pandemic in East Africa. Entomologia Experimentalis et Applicata 99: 13–33.

[23] Li H, Durbin R (2009) Fast and accurate short read alignment with Burrows-Wheeler transform. Bioinformatics 25: 1754–1760. 19451168 10.1093/bioinformatics/btp324 PMC2705234

[24] Wang Z, Gerstein M, Snyder M (2009) RNA-Seq: a revolutionary tool for transcriptomics. Nature Review Genetics 10: 57–63. 10.1038/nrg2484 PMC2949280 19015660

[25] Kanehisa M, Goto S, Sato Y, Furumichi M, Tanabe M (2012) KEGG for integration and interpretation of large-scale molecular data sets. Nucleic Acids Research 40: 109–114. 10.1093/nar/gkr988 PMC3245020 22080510

[26] Huang da W, Sherman BT, Lempicki RA (2009) Systematic and integrative analysis of large gene lists using DAVID bioinformatics resources. Nature Protocols 4: 44–57. 19131956 10.1038/nprot.2008.211

[27] Huang DW, Sherman BT, Lempicki RA (2009) Bioinformatics enrichment tools: paths toward the comprehensive functional analysis of large gene lists. Nucleic Acids Research 37: 1–13. 19033363 10.1093/nar/gkn923 PMC2615629

[28] McSween S, Walker T, Salegua V, Pitoro R (2006) Economic impact on food 438 security of varietal tolerance to cassava brown streak disease in costal Mozambique. In: Series RP, editor. Mozambican Institute of Agricultural Research, Mozambique. 40.

[29] Palukaitis P, Carr JP (2008) Plant resistance responses to viruses. Journal of Plant Pathology 90: 153–171.

[30] Gedil M, Kumar M, Igwe D (2012) Isolation and characterization of resistant gene analogs in cassava, wild *Manihot* species, and castor bean (*Ricinus communis*). African Journal of Biotechnology 11: 15111–15123.

[31] Robaglia C, Caranta C (2006) Translation initiation factors: a weak link in plant RNA virus infection. Trends in Plant Science 11: 40–45. 16343979 10.1016/j.tplants.2005.11.004

[32] Vanderschuren H, Moreno I, Anjanappa RB, Zainuddin IM, Gruissem W (2012) Exploiting the combination of natural and genetically engineered resistance to cassava mosaic and cassava brown streak viruses impacting cassava production in Africa. PLoS ONE 7: e45277. 23049780 10.1371/journal.pone.0045277 PMC3458115

[33] Yadav JS, Ogwok E, Wagaba H, Patil BL, Bagewadi B, et al (2011) RNAi-mediated resistance to Cassava brown streak Uganda virus in transgenic cassava. Molecular Plant Pathology 12: 677–687. 21726367 10.1111/j.1364-3703.2010.00700.x PMC6640337

[34] Mkamillo GS, Jeremiah SC (2005) Current status of cassava improvement programme in Tanzania. African Crop Science Conference Proceedings 7: 1311–1314.

[35] Jones JDG, Dangl JL (2006) The plant imminue system. Nature 444: 323–329. 17108957 10.1038/nature05286

[36] Zenoni S, Ferrarini A, Giacomelli E, Xumerle L, Fasoli M, et al (2010) Characterization of transcriptional complexity during berry development in *Vitis vinifera* using RNA-Seq. Plant Physiology 152: 1787–1795. 20118272 10.1104/pp.109.149716 PMC2850006

[37] Olsen AN, Ernst HA, Leggio LL, Skriver K (2005) NAC transcription factors: structurally distinct, functionally diverse. Trends in Plant Science 10: 79–87. 15708345 10.1016/j.tplants.2004.12.010

[38] Ren T, Qu F, Morris TJ (2000) HRT gene function requires interaction between a NAC protein and viral capsid protein to confer resistance to turnip crinkle virus. Plant Cell 12: 1917–1926. 11041886 10.1105/tpc.12.10.1917 PMC149129

[39] Xie Q, Sanz-Burgos AP, Guo H, Garcia JA, Gutierrez C (1999) GRAB proteins, novel members of the NAC domain family, isolated by their interaction with a geminivirus protein. Plant Molecular Biology 39: 647–656. 10350080 10.1023/a:1006138221874

[40] Cooper JI, Jones AT (1983) Responses of plants to viruses: proposals for the use of terms. Phytopathology 73: 127–128.

[41] Kang BC, Yeam I, Jahn MM (2005) Genetics of plant virus resistance. Annual Reviews of Phytopathology 43: 581–621. 10.1146/annurev.phyto.43.011205.141140 16078896

[42] Nakashita H, Yasuda M, Nitta T, Asami T, Fujioka S, et al (2003) Brassinosteroid functions in a broad range of disease resistance in tobacco and rice. Plant Journal 33: 887–98. 12609030 10.1046/j.1365-313x.2003.01675.x

[43] Mbanzibwa DR, Tian YP, Tugume AK, Mukasa SB, Tairo F, et al (2009) Genetically distinct strains of Cassava brown streak virus in the Lake Victoria basin and the Indian Ocean coastal area of East Africa. Archives of Virology 154: 353–359. 19184340 10.1007/s00705-008-0301-9

