# Supplementary material for: Transcriptional Response of Virus-Infected Cassava and Identification of Putative Sources of Resistance for Cassava Brown Streak Disease
Source: PLoS One. 2014 May 20;9(5):e96642. doi: 10.1371/journal.pone.0096642 (PMC4028184; doi:10.1371/journal.pone.0096642)
Supplement: Table S1 — Sequence of primers used in this study for real time PCR. (DOCX) [file pone.0096642.s001.docx]

Table S1: Primers used for gene expression quantification by real time PCR.

| gene | cassava gene id | TAIR homolog | primer sequence (5'->3') | reference |
| --- | --- | --- | --- | --- |
| RubiscoL |  |  | CTTTCCAAGGCCCGCCTCA | Nassuth et al., 2000 |
|  |  |  | CATCATCTTTGGTAAAATCAAGTCCA |  |
| Ribosomal protein (L2) |  |  | TGGTGTTGCCATGAACCCTGTAGA | Nicot et al., 2005 |
|  |  |  | CGACCAGTCCTCCTTGCAGC |  |
| NAC domain protein homologs | cassava4.1_026167m | AT5G22380.1 | GCCCCACTTGGACGAAAAAC | This study |
|  |  |  | GCGTTGGATTTGCACCAGTT |  |
|  | cassava4.1_026590m | AT5G61430.1 | CATGAATACAGGCTGGACGGT |  |
|  |  |  | ACACCCTGCAAATGACCCAT |  |
|  | cassava4.1_015961m | AT4G35580.3 | ATGCAGTGGCCGACAAACTA |  |
|  |  |  | GCATCAGCTACGTACCCCTT |  |
|  | cassava4.1_028212m | AT4G35580.3 | CCGAACCCAGAGGGATTTCG |  |
|  |  |  | CACGGTACTCGATCCACCTG |  |
|  | cassava4.1_011029m | AT5G46590.1 | GTGTCATCACTCAAGCCCCA |  |
|  |  |  | ATCAGGCGAAACCCATAGGC |  |
|  | cassava4.1_023870m | AT4G35580.2 | TGGTGGAGGTAATTCGCCAAA |  |
|  |  |  | AGCTCGACTTCCTGTTGTGG |  |

References:

Nassuth A, Pollari E, Helmeczy K, Stewart S, Kofalv SA, 2000. Improved RNA extraction and one-tube RT-PCR assay for simultaneous detection of control plant RNA plus several viruses in plant extracts. Journal of Virological Methods 90: 37–49.

Nicot N, Hausman JF, Hoffmann L, Evers D (2005) Housekeeping gene selection for real-time RT-PCR normalization in potato during biotic and abiotic stress. Journal of Experimental Botany 56: 2907–2914.
